# Carbapenem-resistant *Enterobacterales* infection and colonization in patients with severe burns: a retrospective cohort study in a single burn center

**DOI:** 10.1186/s13756-025-01514-9

**Published:** 2025-01-29

**Authors:** Myongjin Kim, Kibum Jeon, Dohern Kym, Jinsun Jung, Yu Jin Jang, Seung Beom Han

**Affiliations:** 1 Department of Surgery and Critical care, Burn Center, Hallym University Hangang Sacred Heart Hospital, Seoul, Korea; 2 Infection Control and Prevention Unit, Hallym University Hangang Sacred Heart Hospital, Seoul, Korea; 3 Department of Laboratory medicine, Hallym University Hangang Sacred Heart Hospital, Seoul, Korea; 4 Department of Pediatrics, College of Medicine, The Catholic University of Korea, 222, Banpo-daero, Seocho-gu, Seoul Republic of Korea

**Keywords:** Carbapenem-resistant *Enterobacteriaceae*, Carbapenemase, Infections, Burns, Korea

## Abstract

**Background:**

Clinical characteristics and outcomes of carbapenem-resistant *Enterobacterales* (CRE) infection and colonization have rarely been reported in patients with severe burns, who are prone to severe bacterial infections. This study aimed to evaluate clinical characteristics and outcomes of CRE infection and colonization in patients with severe burns.

**Methods:**

The characteristics of 106 episodes of CRE acquisition (infection or colonization) in 98 patients with severe burns were evaluated by a retrospective medical record review. The duration of rectal CRE colonization and its associated factors were determined in the survived patients.

**Results:**

Five (4.7%) of the CRE acquisitions were identified on admission, and the remaining 101 (95.3%) were identified at a median of 11 days (range 2–75 days) after admission. *Klebsiella pneumoniae* represented 73.6% of the isolated CRE strains, and carbapenemase-producing CRE (CP-CRE) were identified in 70.8% of the isolates. Mortality was associated with an abbreviated burn severity index (ABSI) score ≥ 10 (*p* < 0.001) and previous carbapenem-resistant bacterial acquisition (protective, *p* = 0.010). For the 58 episodes of CRE acquisition in the survived patients, eradication of rectal CRE colonization was identified in 39 (67.2%) at a median of 64 days (range 10–434 days) after acquisition. CP-CRE strains were associated with prolonged rectal CRE colonization (*p* < 0.001).

**Conclusions:**

The characteristics of CRE infection and colonization in patients with severe burns were similar to those in general critical patients. Enhanced infection prevention and control measures should be considered for patients with severe burns of an ABSI score ≥ 10 and those with CP-CRE.

## Background

The risk for invasive bacterial infections is high in patients with severe burns due to damaged cutaneous physical barriers and immunocompromised state arising from the hyper-metabolic state [1, 2]. Moreover, there is an increased risk for nosocomial infections caused by multidrug-resistant (MDR) bacteria because of usual administration of broad spectrum antibiotics for suspicious sepsis, which is clinically undifferentiated from dysregulated hyper-metabolic state, and prolonged hospitalization until the recovery of the damaged physical barrier [1, 2]. *Acinetobacter baumannii*,* Pseudomonas aeruginosa*, and *Enterobacterales* are major pathogens of invasive infections in patients with severe burns, and MDR strains are increasing among them [2]. Carbapenem-resistant *Enterobacterales* (CRE) is a major MDR pathogen, along with carbapenem-resistant *A. baumannii* and *P. aeruginosa*, for which there is urgent need for developing new active antibiotic agents [3]. However, the clinical characteristics of CRE infection and colonization in patients with severe burns accompanying extensive disruption of the protective physical barrier were rarely reported [4–6].

Effective antibiotics on CRE are limited, and this leads to prolonged hospitalization, higher medical cost, and higher mortality [7]. Especially in Korea, the limitation on the use of newly developed antibiotics that are effective on CRE infections yields increased concern in real-life clinical settings [7]. Therefore, infection prevention and control (IPC) measures for CRE should be performed beyond early diagnosis and treatment of CRE infections, and epidemiological and clinical characteristics of CRE infection and colonization should be investigated to establish appropriate IPC measures for patients with severe burns.

In this study, we investigated epidemiological and clinical characteristics of patients with CRE acquisition (infection or colonization) during hospitalization in the burn intensive care unit (BICU) for acute burn care. In addition, the duration of CRE colonization and factors for prolonged CRE colonization were also evaluated.

## Methods

### Subject and study design

Among the patients hospitalized for acute burn care in the BICU of Hallym University Hangang Sacred Heart Hospital (Seoul, Korea) between January 2020 and December 2022, those in whom CRE was identified in bacterial cultures of any kinds of clinical samples, including surveillance samples, were recruited. CRE infection and colonization were not distinguished, and all patients with CRE acquisition were included in this study. Their medical records were retrospectively reviewed to collect demographic, clinical, and microbiological data. Demographic data included sex and age, and clinical data included underlying diseases; burn characteristics; BICU duration; use of invasive devices (central venous catheter [CVC], urethral catheter, endotracheal tube), administered antibiotics, and acquisition of other carbapenem-resistant Gram-negative bacteria (CR-GNB) during hospitalization; and in-hospital death. Microbiological data included isolated CRE species and carbapenemase types, as well as their antibiotic resistance rates. If a different species of CRE was identified after eradication of the initial species, it was considered a separate episode of CRE acquisition and included in the study analysis. For repeat identifications of the same CRE species with an identical carbapenemase during the same hospitalization period, only the first episode was included in the study analysis. CRE acquisition episodes were divided into two groups according to survival of the patient during the CRE acquisition period: survived and deceased groups. The investigated data were compared between the two groups to identify significant factors associated with death during CRE acquisition.

For the survived group, follow-up rectal swab culture results were reviewed until December 31, 2023. The duration of CRE colonization was determined in patients in whom CRE was identified on a rectal swab culture and at least one follow-up rectal swab culture was performed. CRE eradication was defined when three consecutive rectal swab cultures performed at an interval of ≥ 3 days were negative for CRE. The day when the third negative sample was collected was defined as a CRE eradication day. The patients were categorized into eradication and persistence groups according to CRE eradication status. The duration of CRE colonization was calculated from the day when the first positive sample for CRE was collected to the CRE eradication day in the eradication group, and to the day when the last rectal swab culture was performed in the persistence group. The investigated data were compared between the eradication and persistence groups to identify significant factors associated with CRE eradication. This study was approved by the Institutional Review Board of Hallym University Hangang Sacred Heart Hospital with a waiver for informed consent (approval number: HG2023-026).

### Microbiological tests

CRE surveillance cultures for rectal swab samples were performed using a commercially available chromogenic agar (CHROMagar™ KPC, Chromagar, Paris, France). Cultures of clinical samples and antibiotic susceptibility tests were performed using a BD Phoenix M50 automated system (Becton, Dickinson and Company, NJ, USA). CRE was defined as isolated *Enterobacterales* not susceptible to one or more carbapenems: minimal inhibitory concentration (MIC) > 0.5 µg/mL for ertapenem and > 1 µg/mL for doripenem, imipenem, and meropenem. The presence and type (KPC, IMP, NDM, VIM, OXA-48) of carbapenemase were determined using a commercially available lateral flow immunoassay kit (NG Test^®^ CARBA 5 NG Biotech, Guipry, France). If negative for carbapenemase, a multiplex polymerase chain reaction test for *KPC*,* IMP*,* GES*,* NDM*,* VIM*, and *OXA-48* genes was additionally performed using a PANA RealTyper™ CRE Kit (HLB PANAGENE Co., LTD., Daejeon, Korea). Isolates that were positive for either of two carbapenemase tests were defined as carbapenemase-producing CRE (CP-CRE), and the remaining isolates were defined as non-CP-CRE.

### Infection prevention and control strategies

In our hospital, standard and contact precautions are applied to all patients during their BICU stay. On admission, an active surveillance culture for CRE using a rectal swab is performed, and weekly surveillance cultures are repeated during the BICU stay. If CRE is identified, the patient is isolated or cohorted in a separate room in the BICU (two isolation rooms and three two-bed rooms) as soon as possible, a surveillance culture using a rectal swab is performed for the adjacent patients, and enhanced environmental cleaning is performed. Following the identification of rectal CRE colonization, follow-up rectal swab cultures are performed at an interval of ≥ 3 days until CRE eradication. When CRE eradication is confirmed during the BICU stay, the patient is released from isolation or cohorting. If a patient is transferred from the BICU to a general ward before CRE eradication, the patient is placed in isolation or cohorting in the general ward. Patients with CRE colonization at the time of hospital discharge are preemptively isolated or cohorted upon re-admission.

### Statistical analysis

Between the survived and deceased groups, continuous variables were compared using student’s t-test or Mann-Whitney U test according to normality, and categorical variables were compared using a chi-square test. Significant variables in a univariate analysis were included into a multivariate analysis using a binary logistic regression test to determine independent risk factors for death during CRE acquisition. For the survived group, clinical variables were compared between the eradication and persistence groups, and a multivariate Cox proportional hazard regression analysis was performed to identify independent factors associated with the duration of CRE colonization. Statistical analyses were performed using the SPSS 27 program (IBM, Armonk, NY), and statistical significance was defined as a two-tailed *p*-value < 0.05.

## Results

### Characteristics of CRE acquisition episodes

**Fig. 1:**
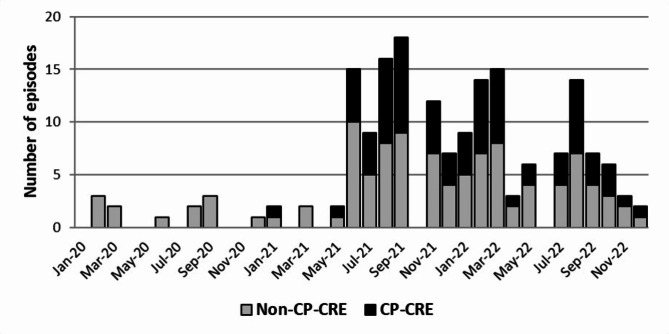
Epidemic curve for CRE infection/colonization

During the study period, 106 episodes of CRE acquisition were identified in 98 patients with severe burns. Eight patients each experienced two episodes of CRE acquisition caused by different CRE species. CRE acquisition has increased from June 2021 (Fig. 1). Most (*n* = 74, 75.5%) of the included 98 patients experienced flame burns, and inhalation injury was accompanied in 48 (49.0%) patients. The median percentage of total body surface area (%TBSA) burn was 42% (range 1–97%), and the median abbreviated burn severity index (ABSI) score was 9 (range 5–15).

CRE acquisition was identified by active surveillance cultures upon admission in five (4.7%) episodes, surveillance cultures after in-hospital exposure to other CRE-acquired patients in eight (7.5%) episodes, weekly surveillance cultures in 41 (38.7%) episodes, and clinical sample cultures performed as a fever study in 52 (49.1%) episodes (Table 1). Among the five patients with CRE colonization on BICU admission, one patient had concurrent bacteremia caused by KPC-positive *Klebsiella pneumoniae*. The other three patients with KPC-positive *K. pneumoniae* colonization, along with the remaining patient with NDM-positive *Enterobacter cloacae* colonization, did not develop a CRE infection during their BICU stay. For the 101 episodes in 93 patients identified after admission, CRE acquisition was identified at a median of 11 days (range 2–75 days) after admission to the BICU. One patient was transferred from another hospital after undergoing burn wound excision and grafting, while seven patients developed CRE acquisition before receiving surgical intervention for their burn wounds. The remaining 85 patients underwent their first surgical intervention for burn wounds at a median of 2 days (range 0–13 days) after BICU admission. For all episodes of them, the patient had two or more invasive devices and had received antibiotic therapy, and 97.0% (98/101) of episodes occurred when other CRE-acquired patients were present in the BICU. A total of 34 (32.1%) deaths occurred during CRE acquisition, and 13 (38.2%) of them were attributable to CRE infection. *K. pneumoniae* represented 73.6% (*n* = 78) of the isolated CRE strains, and CP-CRE was isolated in 75 (70.8%) episodes: 73 (97.3%) strains with KPC and two (2.7%) strains with NDM. The acquisition of KPC-positive CP-CRE also has increased from June 2021 along with the acquisition of CRE (Fig. 1).

In 64 (60.4%) of 106 episodes of CRE acquisition, CRE was initially identified as colonization. Of these, 46 (43.4%) episodes continued as colonization during BICU care, while CRE was subsequently cultured from clinical samples in the remaining 18 (17.0%) episodes: 12 from wounds, 10 from blood, four from respiratory secretions, and one from urine. CRE was identified simultaneously in two or more samples in eight (44.4%) of these episodes, and other antibiotic-resistant bacteria were detected alongside CRE in the same sample in another eight (44.4%) episodes. In 42 (39.6%) episodes, CRE was initially identified from cultures of rectal swabs and other clinical samples simultaneously: 28 from wounds, 27 from blood, and 13 from respiratory secretions. CRE was identified simultaneously in two or more samples in 20 (47.6%) of these episodes, and other antibiotic-resistant bacteria were detected alongside CRE in the same sample in 32 (76.2%) episodes.

### Comparison between the survived and deceased groups

None of the patients in the deceased group experienced recurrent CRE acquisition or CRE eradication before death. The mortality rate was highest during 2021 when the CRE acquisition began to increase (*p* = 0.009) (Table 1). The deceased group showed significantly higher %TBSA burn (*p* < 0.001) and ABSI score (*p* < 0.001), as well as higher frequencies of endotracheal intubation (*p* = 0.001) and renal replacement therapy (*p* = 0.013) than the survived group (Table 1). CRE acquisition occurred earlier after BICU admission in the deceased group than in the survived group (*p* < 0.001). The deceased group showed significantly lower frequencies of CR-GNB acquisition (*p* = 0.002) and fluoroquinolone (*p* = 0.001), colistin (*p* = 0.009), and glycopeptide (*p* = 0.001) administration after admission (Table 1). The species distribution and antibiotic resistance rates of the isolated CRE strains were comparable between the two groups; however, CP-CRE strains were more frequent in the deceased group than in the survived group (*p* = 0.007) (Table 1).

**Table 1:** Comparison of clinical characteristics between the survived and deceased groups

Factor	**Survived group**	**Deceased group**	*p* value
	**(N = 72)**	**(N = 34)**	
Male sex	59 (81.9)	26 (76.5)	0.509
Age, years, mean ± SD	51.9 ± 18.8	57.4 ± 19.1	0.171
Year			0.009
2020	12 (16.7)	0 (0.0)	
2021	26 (36.1)	21 (61.8)	
2022	34 (47.2)	13 (38.2)	
Admission from other hospitals	10 (13.9)	4 (11.8)	> 0.99
Identification of CRE			0.182
On admission	3 (4.2)	2 (5.9)	
On contact surveillance	5 (6.9)	3 (8.8)	
On regular screening	33 (48.6)	8 (23.5)	
On infection suspicion	31 (40.3)	21 (61.8)	
CCI ≥ 3 points	22 (30.6)	13 (38.2)	0.433
Burn type			0.683
Flame	54 (75.0)	26 (76.5)	
Scald	7 (9.7)	4 (11.8)	
Electrical	6 (8.3)	2 (5.9)	
Contact	2 (2.8)	2 (5.9)	
Chemical	3 (4.2)	0 (0.0)	
Inhalation injury	26 (36.1)	19 (55.9)	0.055
TBSA burn, %, mean ± SD	37.1 ± 17.8	53.2 ± 23.5	< 0.001
ABSI score ≥ 10	17 (23.6)	27 (79.4)	< 0.001
Central venous catheter	68 (94.4)	34 (100.0)	0.303
Urethral catheter	72 (100.0)	34 (100.0)	NA
Endotracheal intubation	38 (52.8)	29 (85.3)	0.001
Continuous renal replacement therapy	1 (1.4)	5 (14.7)	0.013
BICU days before CRE identification, median (range)	12 (0–75)	6 (0–37)	< 0.001
Previous CR-GNB acquisition	52 (72.2)	14 (41.2)	0.002
Antibiotic use before CRE acquisition			
Non-anti-pseudomonal β-lactams	9 (12.5)	0 (0.0)	0.055
Piperacillin/tazobactam	64 (88.9)	31 (91.2)	> 0.99
Anti-pseudomonal cephalosporins	10 (13.9)	1 (2.9)	0.101
Carbapenems	26 (36.1)	6 (17.6)	0.053
Aminoglycosides	14 (19.4)	3 (8.8)	0.164
Fluoroquinolones	22 (30.6)	1 (2.9)	0.001
Colistin	35 (48.6)	7 (20.6)	0.006
Tigecycline or minocycline	7 (9.7)	0 (0.0)	0.094
Glycopeptides	46 (63.9)	10 (29.4)	< 0.001
Positive sample for CRE			0.28
Rectal swab only	24 (33.3)	7 (20.6)	
Clinical sample only	13 (18.1)	5 (14.7)	
Both	35 (48.6)	22 (64.7)	
CP-CRE identification	45 (62.5)	30 (88.2)	0.007
Bacteria species			0.924
* Klebsiella pneumoniae*	51 (70.8)	27 (79.4)	
* Klebsiella aerogenes*	8 (11.1)	3 (8.8)	
* Escherichia coli*	5 (6.9)	1 (2.9)	
* Enterobacter cloacae*	4 (5.6)	2 (5.9)	
* Serratia marcescens*	2 (2.8)	1 (2.9)	
* Proteus mirabilis*	1 (1.4)	0 (0.0)	
* Providencia stuartii*	1 (1.4)	0 (0.0)	
Antibiotic resistance rate			
Ampicillin	69 (95.8)	34 (100.0)	0.55
Amoxicillin/clavulanate	65 (90.3)	32 (94.1)	0.715
Piperacillin/tazobactam	63 (87.5)	30 (88.2)	> 0.99
Caftazidime	63 (87.5)	33 (97.1)	0.163
Cefepime	64 (88.9)	32 (94.1)	0.496
Meropenem^a^	54 (91.5)	32 (94.1)	> 0.99
Amikacin	2 (2.8)	1 (2.9)	> 0.99
Ciprofloxacin	58 (80.6)	31 (91.2)	0.164
Colistin^b^	36 (62.1)	19 (57.6)	0.673
Tigecycline^c^	50 (71.4)	28 (82.4)	0.227
Trimethoprim/sulfamethoxazole	55 (76.4)	30 (88.2)	0.153

*SD* standard deviation, *CRE* carbapenem-resistant *Enterobacterales*, *CCI* Charlson comorbidity index, *TBSA* total body surface area, *ABSI* abbreviated burn severity index, *NA* not available, *BICU* burn intensive care unit, *CR-GNB* carbapenem-resistant Gram-negative bacteria, *CP-CRE* carbapenemase-producing CRE. ^a^ Antibiotic susceptibility was tested in 59 and 34 episodes in the survived and deceased groups, respectively. ^b^ Antibiotic susceptibility was tested in 58 and 33 episodes in the survived and deceased groups, respectively. ^c^ Antibiotic susceptibility was tested in 70 and 34 episodes in the survived and deceased groups, respectively

.

Considering the associations between significant factors identified in a univariate analysis, ABSI score was included in multivariate analysis among factors associated with burn severity including %TBSA burn, ABSI score, endotracheal intubation, and renal replacement therapy. The chances for infection by MDR pathogens and administration of antibiotics against MDR pathogens increase with the length of BICU duration; therefore, CR-GNB acquisition was included in multivariate analysis among BICU duration; CR-GNB acquisition; and administration of fluoroquinolone, colistin, and glycopeptide. A multivariate analysis for ABSI score, CR-GNB acquisition, and CP-CRE showed that ABSI score ≥ 10 (odds ratio 13.549, 95% confidence interval 4.509–40.711, *p* < 0.001) and CR-GNB acquisition (odds ratio 0.242, 95% confidence interval 0.082–0.716, *p* = 0.010) were significantly associated with death (Table 2).

**Table 2:** Multivariate analysis for factors associated with mortality

Factor	Odds ratio	95% CI	*p* value
ABSI score ≥ 10	13.549	4.509–40.711	< 0.001
CR-GNB acquisition	0.242	0.082–0.716	0.010
CP-CRE identification	2.731	0.714–10.447	0.142

*CI* confidence interval, *ABSI* abbreviated burn severity index, *CR-GNB* carbapenem-resistant Gram-negative bacteria, *CP-CRE* carbapenemase-producing carbapenem-resistant *Enterobacterales*

### Duration of rectal CRE colonization

For the 72 episodes in the survived group, rectal swab cultures were negative for CRE in 13 (18.1%) episodes, and follow-up rectal swab cultures were not performed in one (1.4%) patient who was transferred to another hospital. CRE colonization duration and factors for CRE eradication were determined in the remaining 58 (80.6%) episodes. During a median of 68 days (range 10–769 days) of follow-up, CRE were eradicated in 39 (67.2%) episodes at a median of 64 days (range 10–434 days) after acquisition. Among the 39 episodes in the eradication group, CRE eradication occurred during the BICU stay in nine episodes, and during general ward care following BICU care before discharge in 12 episodes. The remaining 18 episodes of CRE eradication occurred during re-admission care for rehabilitation. Of the 19 episodes in the persistence group, five patients were transferred to other hospitals while colonized with CRE. Nine patients continued outpatient care after discharge with CRE colonization, without follow-up rectal swab cultures or re-admission being performed. The remaining five patients showed persistent CRE colonization upon re-admission for rehabilitation during the study period. Younger age (*p* = 0.001), lower Charlson comorbidity index (CCI) score (*p* = 0.004), higher %TBSA burn (*p* = 0.009), and non-CP-CRE (*p* = 0.005) were associated with CRE eradication in a univariate analysis (Table 3).

**Table 3:** Comparison of clinical characteristics between eradication and persistence groups

Factor	**Eradication group**	**Persistence group**	*p* value
	**(N = 39)**	**(N = 19)**	
Follow-up days, median (range)	64 (10–434)	88 (23–769)	0.129
Male sex	33 (84.6)	12 (63.2)	0.095
Age, years, mean ± SD	46.1 ± 18.4	63.9 ± 16.3	0.001
Year			0.005
2020	9 (23.1)	0 (0.0)	
2021	16 (41.0)	4 (21.1)	
2022	14 (35.9)	15 (78.9)	
Identification of CRE			0.573
On admission	1 (2.6)	2 (10.5)	
On contact surveillance	3 (7.7)	2 (10.5)	
On regular screening	23 (59.0)	9 (47.4)	
On infection suspicion	12 (30.8)	6 (31.6)	
CCI ≥ 3 points	8 (20.5)	11 (57.9)	0.004
Burn type			0.101
Flame	30 (76.9)	13 (68.4)	
Scald	4 (10.3)	2 (10.5)	
Electrical	4 (10.3)	0 (0.0)	
Contact	0 (0.0)	2 (10.5)	
Chemical	1 (2.6)	2 (10.5)	
Inhalation injury	14 (35.9)	6 (31.6)	0.745
TBSA burn, %, mean ± SD	41.8 ± 16.7	28.8 ± 17.7	0.009
ABSI score ≥ 10 points	9 (23.1)	6 (31.6)	0.533
Central venous catheter	37 (94.9)	17 (89.5)	0.591
Foley catheter	39 (100.0)	19 (100.0)	NA
Endotracheal intubation	19 (48.7)	9 (47.4)	0.923
Renal replacement therapy	1 (2.6)	0 (0.0)	> 0.99
BICU days before CRE identification, median (range)	12 (0–75)	12 (0–57)	0.797
BICU days after CRE identification, median (range)	17 (1–169)	30 (0–105)	0.211
Positive sample for CRE			0.147
Rectal swab only	18 (46.2)	5 (26.3)	
Rectal swab and clinical sample	21 (53.8)	14 (73.3)	
CP-CRE identification	26 (66.7)	19 (100.0)	0.005
Bacteria species			0.717
*K. pneumoniae*	31 (79.5)	17 (89.5)	
*K. aerogenes*	4 (10.3)	2 (10.5)	
*E. coli*	1 (2.6)	0 (0.0)	
*E. cloacae*	2 (5.1)	0 (0.0)	
*S. marcescens*	1 (2.6)	0 (0.0)	

*SD* standard deviation, *CRE* carbapenem-resistant *Enterobacterales*, *CCI* Charlson comorbidity index, *TBSA* total body surface area, *ABSI* abbreviated burn severity index, *NA* not available, *BICU* burn intensive care unit, *CP-CRE* carbapenemase-producing CRE

**Fig. 2:**
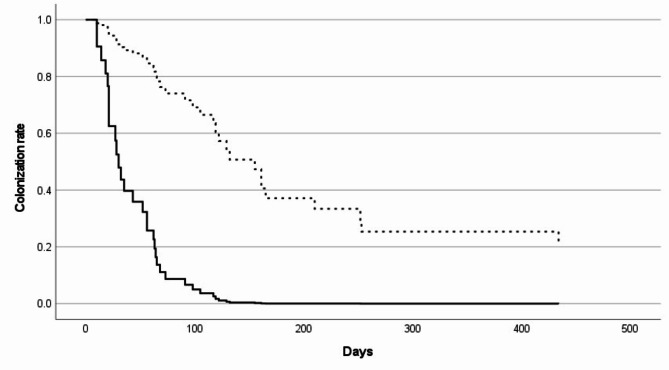
Rectal colonization rate after CRE acquisition

Except for age, which is included in the calculation of a CCI score, CCI score, %TBSA burn, and CP-CRE were included in the Cox proportional hazard regression analysis, and CP-CRE showed a significant negative association with CRE eradication (*p* < 0.001; Table 4; Fig. 2). All of the non-CP-CRE (*n* = 13) were eradicated at a median of 27 days (range 10–117 days) after acquisition, and 57.8% (26/45) of the CP-CRE were eradicated at a median of 102 days (range 18–434 days) after acquisition.

**Table 4:** Result of the multivariate Cox regression analysis for CRE eradication

Factor	Hazard ratio	95% CI	*p* value
CCI ≥ 3 points	0.571	0.243–1.344	0.200
TBSA	1.014	0.992–1.037	0.222
CP-CRE	0.123	0.054–0.281	< 0.001

*CRE* carbapenem-resistant *Enterobacterales*, *CI* confidence interval, *CCI* Charlson comorbidity index, *TBSA* total body surface area, *CP-CRE* carbapenemase-producing CRE

## Discussion

In this study, the epidemiological and clinical characteristics of CRE-acquired patients among those with severe burns were consistent with those reported in Korean critical patients: *K. pneumoniae* are the most common CRE species and > 70% of the CRE strains produced carbapenemase, most frequently KPC [8]. However, the mortality rate during CRE acquisition in patients with severe burns was lower than that (42.3%) reported in a meta-analysis for hospitalized patients with CRE infections [9].

Although patients with severe burns are prone to acquire severe invasive infections, younger age and lower frequency of underlying diseases in patients with severe burns in this study than those of other critical patients might have impacted the mortality rate. The mean age of the included patients in this study was 54.1 years and their median CCI score was 2 (range 0–8) with a CCI score less than 3 in 64.3% of them. In previously reported studies on CRE infection and colonization, the means of age and CCI score of the included general critical patients were 62–72 years and 4, respectively [10–14]. Therefore, we can expect greater impact of IPC measures for preventing CRE acquisition and early diagnosis and treatment of CRE infection on improving outcomes in relatively younger and healthier patients with severe burns than in general critical patients.

The mortality in this study was significantly associated with burn severity. Considering that the more severe is a burn, the earlier invasive infections occur and the more numerous are infections by MDR pathogens [1], this result was not surprising. Clinical severity was most significantly associated with mortality in patients with CRE infections in previous studies, along with inappropriate antibiotic therapy and infection site [15]. Most strains of *A. baumannii* and *P. aeruginosa*, which are major pathogens of burn infections in our hospital, are carbapenem-resistant [16]. The deceased group in this study showed lower acquisition rate of such CR-GNB and lower administration rate of fluoroquinolone and colistin, which are used for treating CR-GNB infections replacing β-lactam agents, before CRE acquisition than the survived group. The deceased group acquired CRE earlier during BICU stay than the survived group, suggesting that CRE acquisition in the early phase of BICU care, prior to acquisition of CR-GNB, was associated with grave outcomes. Therefore, enhanced IPC measures for preventing CRE acquisition should be considered immediately after admission to the BICU in patients with ABSI score ≥ 10. Several IPC measures effective for preventing CRE acquisition, including contact precaution, isolation and cohorting, and active surveillance, have been performed in our hospital [17]. Therefore, enhanced environmental cleaning and staff education can be additionally implemented to control CRE spread [17]. However, CRE has been rarely identified in environmental sample cultures in the BICU of our hospital, and previously reported identification rates of CRE in environmental samples were less than 10% [11, 18, 19]. Therefore, ongoing CRE acquisition among patients in the BICU in our hospital suggests the important role of transmission vectors between CRE-acquired and CRE-naïve patients, such as healthcare workers and medical equipment. Staff cohorting, e.g., caring for only CRE-acquired patients, is not easy to perform in real-life clinical settings with limited numbers of healthcare workers. Enhanced hand hygiene, contact precautions, and equipment reprocessing should be preferentially applied.

Whether CP-CRE cause higher mortality than non-CP-CRE is controversial [20–22]. A previous study showed that CP-CRE caused higher mortality than non-CP-CRE in patients with bacteremia; however, CP-CRE had higher resistance rates against antibiotics other than carbapenems and higher MIC for carbapenems than non-CP-CRE in that study [20]. Increased mortality due to CP-CRE infection was not observed in other patients with various types of infections including bacteremia [21], and a Korean study did not reveal increased mortality due to CP-CRE infection compared with non-CP-CRE infection in patients with bacteremia under comparable antibiotic resistance rates [22]. In the present study, there was no significant difference in antibiotic resistance rates and mortality in a multivariate analysis between CP-CRE and non-CP-CRE.

The duration of rectal CRE colonization varied according to patient characteristics, definition of CRE eradication, and applied IPC measures in previous studies, and a colonization rate of 55.2% at 6 months after CRE acquisition was reported in a meta-analysis [23]. In the present study, two-thirds of the survived group achieved rectal CRE eradication at a median of 64 days after CRE acquisition, while rectal colonization continued for longer than 2 years in one patient. CP-CRE strains were significantly associated with prolonged colonization, and resultantly, CP-CRE showed a lower eradication rate and longer duration of rectal colonization than non-CP-CRE. Most previous studies on the duration of rectal CRE colonization were based on CP-CRE colonization; and CP-CRE infection (vs. colonization), re-admission, prolonged antibiotic use, and prolonged hospitalization were associated with prolonged CP-CRE rectal colonization [24–26]. CP-CRE species and the type of carbapenemase showed an impact on the duration of CP-CRE colonization [27]. Therefore, differentiated IPC measures and isolation/cohorting strategies might be necessary for patients with CP-CRE infection or colonization among CRE-acquired patients with consideration for these factors.

This study had some limitations. As we retrospectively reviewed pre-existing medical records, culture studies were not performed systematically. Although weekly rectal swab cultures were performed, clinical sample cultures in patients suspicious of infections were performed according to the attending physician’s preference. Some patients with undiagnosed CRE infection not accompanying rectal colonization could be missed. Information on previous hospitalization and antibiotic use several months before admission to our hospital, which might be associated with CRE acquisition, could not be investigated. Genetic relatedness of the isolated CRE was not determined, and therefore, the exact dynamics of in-hospital transmission of CRE could not be defined. However, most CRE acquisition seemed to result from in-hospital transmission because almost all episodes occurred when other CRE-acquired patients were present in the BICU. Although significant differences in clinical outcomes have been reported between patients with CRE infection and those with CRE colonization [11, 12, 28], these states were not distinguished in the present study. To diagnose catheter-associated bloodstream infection, two separate blood samples should be collected. However, in our patients, only one sample was usually obtained through a CVC due to limited access to peripheral veins caused by extensive skin damages. This made it difficult to differentiate true bloodstream infection from bacterial CVC colonization. Although clinical criteria for diagnosing sepsis associated with local infections in patients with severe burns have been recommended by the American Burn Association, it remains challenging to distinguish between bacteria colonizing burn wounds and those causing invasive infections. This is partly because many hospitals, including ours, perform qualitative rather than quantitative cultures for wound samples. Additionally, pneumonia and lung injuries due to inhalation, pulmonary edema, or acute respiratory distress syndrome may not be easily differentiated in clinical settings [29]. CRE is commonly cultured from multiple clinical samples simultaneously, complicating the differentiation between infection and colonization sites. Other antibiotic resistant-bacteria are also frequently cultured alongside CRE in the same clinical samples, further complicating the distinction between infecting and colonizing pathogens. The culture results from our patients showed a similar pattern. Since CRE colonization is thought to predispose CRE infection, the lack of differentiation between CRE infection and colonization in this study likely had little impact.

## Conclusion

In this study, epidemiological, clinical, and microbiological characteristics of CRE infection/colonization in patients with severe burns were similar to those in general critical patients. Clinical outcomes of CRE-acquired patients were associated with burn severity. Enhanced IPC measures might be necessary for patients acquiring CP-CRE with consideration provided for the association between CP-CRE strains and prolonged CRE colonization.

## Data Availability

No datasets were generated or analysed during the current study.

## Abbreviations

ABSI: Abbreviated burn severity index
BICU: Burn intensive care unit
CCI: Charlson comorbidity index
CP-CRE: Carbapenemase-producing carbapenem-resistant *Enterobacterales*
CRE: Carbapenem-resistant *enterobacterales*
CR-GNB: Carbapenem-resistant gram-negative bacteria
CVC: Central venous catheter
IPC: Infection prevention and control
MDR: Multidrug-resistant
%TBSA: Percentage of total body surface area
